# Structure-based virtual screening identifies potential endogenous ligands of the human bitter taste receptor TAS2R46

**DOI:** 10.1016/j.jbc.2026.113295

**Published:** 2026-06-25

**Authors:** Yuki Nagasato, Keisuke Sanematsu, Yuko Kawabata, Shingo Takai, Noriatsu Shigemura

**Affiliations:** 1 Section of Oral Neuroscience, Graduate School of Dental Science, Kyushu University, Fukuoka, Japan; 2 Oral Health/Brain Health/Total Health Research Center, Graduate School of Dental Science, Kyushu University, Fukuoka, Japan; 3 Research and Development Center for Five-Sense Devices, Kyushu University, Fukuoka, Japan; 4 Dent-craniofacial Development and Regeneration Center, Graduate School of Dental Science, Kyushu University, Fukuoka, Japan

**Keywords:** G protein-coupled receptor (GPCR), molecular dynamics, molecular docking, bitter taste receptor, TAS2R46, drug screening, steroid hormone

## Abstract

The sensing of bitter taste mediated by TAS2Rs serves as a defense mechanism against potentially harmful substances. The expression of TAS2Rs in extra-oral tissues, which are not directly exposed to the external environment, suggests the presence of endogenous ligands and points to TAS2Rs having novel roles as chemical sensors of the internal environment. Here, we performed structure-based screening to identify potential endogenous ligands of TAS2R46, whose structure has recently been determined. Our strategy combined ensemble docking of diverse conformations generated by molecular dynamics simulations of the TAS2R46–strychnine complex with machine learning-based integration of the docking results. This approach improved evaluation metrics compared with single-conformation docking. Screening compounds from the Human Metabolome Database identified nine steroid hormones (including their derivatives) as candidate ligands. Functional assays revealed that eight of these steroids—17-hydroxyprogesterone, testosterone, dihydrotestosterone, dehydroepiandrosterone, androstenedione, corticosterone, deoxycorticosterone, and cortexolone—activated TAS2R46, whereas estrone did not. Boltz-2–based predictions of TAS2R46–steroid complexes and mutational analysis revealed key residues that contribute to both stable steroid binding and subsequent receptor activation. Together, these findings provide new insights into the physiological roles of TAS2R46 in extra-oral tissues and its mechanism of activation, and they establish a broadly applicable framework for ligand prediction across G protein-coupled receptors, including taste receptors.

Bitter taste plays an essential role in preventing the ingestion of potentially toxic substances. Bitter taste receptors are expressed on the apical membrane of taste receptor cells. These receptors belong to the class T G protein-coupled receptor (GPCR) family, known as taste type 2 receptors (TAS2Rs), which number approximately 25 in

humans (1, 2). Structurally, TAS2Rs consist of a short extracellular N-terminus, seven transmembrane α-helices, and an intracellular C-terminus, together forming a relatively unconserved orthosteric binding site on the extracellular side (3). Such variability enables each TAS2R member to acquire ligand specificity (4), thereby allowing the TAS2R family as a whole to respond to a broad range of structurally diverse bitter compounds (5, 6). Activation of the TAS2Rs triggers downstream signal transduction involving co-expressed proteins, such as α-gustducin (7), a specific form of phospholipase C (PLCβ2), transient receptor potential melastatin 5 (TRPM5), and calcium homeostasis modulator 1 (CALHM1)/CALHM3 channel (8, 9, 10).

Recent studies have reported the expression of TAS2Rs in extra-oral tissues, including the intestine, immune cells, testis, pancreas, adipose tissue, airways, heart, and brain (11, 12). This widespread distribution suggests that TAS2Rs may have additional physiological roles beyond the perception of bitter taste in the oral cavity. For example, TAS2Rs expressed in the upper airway have been reported to trigger mucociliary clearance and contribute to host defense (13). However, the physiological roles of TAS2Rs broadly expressed throughout the body remain largely unknown. To activate TAS2Rs expressed in extra-oral tissues that are not directly exposed to the external environment, exogenous bitter compounds must reach sufficient concentrations following absorption from the gastrointestinal tract. Nevertheless, many bitter compounds found in vegetables, such as polyphenols and alkaloids, have low bioavailability (14, 15). These findings suggest the possible existence of endogenous ligands for TAS2Rs.

Screenings of bitter compounds have been facilitated by *in silico* tools, primarily using ligand-based approaches with machine learning algorithms (16). One such model, BitterMatch, not only predicts whether query compounds have a bitter taste but also identifies their potential TAS2R targets (17). Although ligand-based *in silico* tools have shown considerable success, they face inherent limitations that may hinder the discovery of novel ligands—such as biases in training datasets and an inability to fully capture complex receptor–ligand interactions (16).

Structure-based approaches for ligand screening, including molecular docking and molecular dynamics (MD) simulation, have been underutilized for TAS2Rs because of the difficulty in constructing reliable homology models. This difficulty stems from the low sequence identity between TAS2Rs and class A GPCRs with known structures (18), which is in turn due to the historical lack of experimentally determined TAS2R structures. However, recent advances in AI-driven structure modeling (19, 20) and the availability of cryo-EM structures of TAS2R14 (21), TAS2R16 (22), and TAS2R46 (23) have made it possible to generate more accurate structure models, thereby enabling structure-based virtual screening (SBVS) for bitter taste receptors.

Ensemble docking—an approach that takes into account receptor flexibility by using multiple protein conformations—has been employed to overcome the limitations of relying on a single rigid structure, which can compromise predictive performance (24, 25). Recent studies have reported that combining ensemble docking with machine learning, by aggregating multiple docking scores, improves the predictive power of SBVS (26, 27).

The aim of this study was to identify the endogenous ligands of TAS2R46. To this end, we conducted ensemble docking against the Human Metabolome Database (HMDB) (28) using conformations generated by MD simulations and applied machine learning to aggregate the results (Fig. 1). Based on the SBVS, several steroid hormones were identified as candidate compounds and were shown to activate TAS2R46 in a functional assay. These findings provide novel insights into the physiological roles of TAS2Rs expressed in extra-oral tissues.

**Figure 1:**
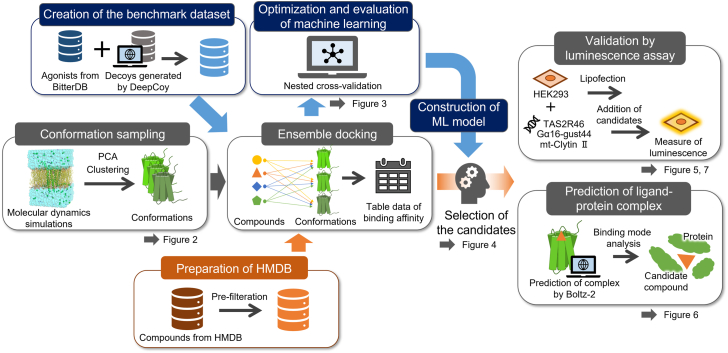
**Overview of the screening and validation workflow.** The screening began with conformation sampling by MD simulations, followed by ensemble docking and construction of a machine learning model. The candidate compounds were selected based on the ensemble docking and prediction results of the constructed machine learning model. TAS2R46-agonistic activity of the candidate compounds was confirmed by functional assay. The structure–activity relationship was analyzed using the TAS2R46–compound complex predicted by Boltz-2.

## Results

### Sampling structural conformations of TAS2R46

**Figure 2:**
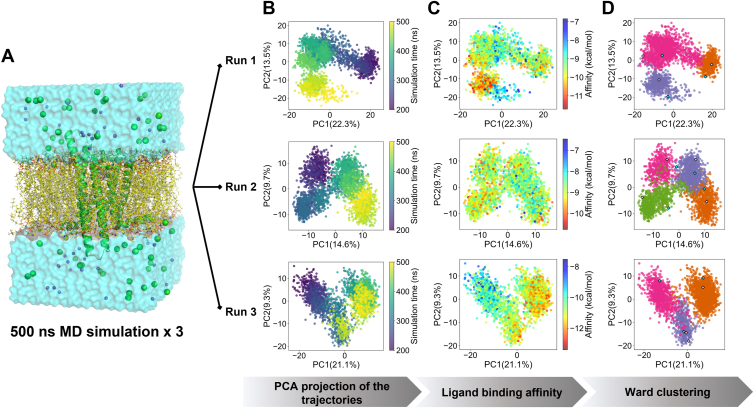
**System used for MD simulations and procedure of sampling diverse conformations from the MD trajectories.***A*, representative simulation system. TAS2R46 was embedded in a POPC bilayer (*yellow wire*) and solvated in 150 mM NaCl. Sodium and chloride ions are shown as *purple* and *green* spheres, respectively. Water molecules are shown as the *cyan* surface. *B–D*, PCA projections of the MD trajectories. The plots are colored according to simulation time (*B*), strychnine binding affinity (*C*), and cluster assignment (*D*). The best and worst models in each cluster are indicated by *pink* and *cyan* diamonds, respectively.

Ensemble docking requires multiple structural conformations; however, only three experimental structures of TAS2R46 are currently available in the Protein Data Bank (PDB). To expand conformational sampling, we conducted three independent 500-ns MD simulations of a TAS2R46–strychnine complex in a palmitoyl-oleoyl-phosphatidylcholine (POPC) lipid bilayer in the absence of G protein (see Experimental procedures). The final 300 ns of each trajectory, during which the RMSD of the Cα atoms had stabilized, were used for analysis (Figs. 2*A* and S1). To capture subtle conformational changes, we focused on 24 binding-site residues involved in ligand recognition (Fig. S2). Principal component analysis (PCA) of the trajectories based on these residues revealed a broad conformational distribution (Figs. 2*B* and S2). Ligand-binding affinities were then evaluated, and cluster analysis further partitioned each trajectory into three or four distinct groups (Figs. 2, *C*, *D* and S3). From each cluster, two representative structures were selected for ensemble docking: one with the highest strychnine-binding affinity (designated as the “best” structure) and another with the lowest binding affinity (designated as the “worst” structure). The selected 20 structures exhibited widely dispersed binding-site conformations in the PCA projection while maintaining high overall structural similarity to the experimental structure (PDB ID: 7XP6; all-Cα RMSD = 0.99–1.29 Å), confirming successful sampling of diverse binding-site conformations (Figs. 2*D* and S4).

Rescoring the binding affinities of strychnine revealed that, in all selected structures, strychnine occupied the orthosteric site but adopted three distinct binding poses (Fig. S5*A*). Although the interaction patterns varied across models and the cryo-EM structure (PDB ID: 7XP6), consistent contacts with the agonism-related residues Trp-88^3.32^ in transmembrane helix (TM) III and Glu-265^7.39^ in TM VII were maintained, except for in one model (Fig. S5*B*) (23). These variations in binding poses and interaction networks underscore the structural heterogeneity of the orthosteric site and suggest multiple feasible binding modes for strychnine in TAS2R46.

### Narrowing down candidate compounds by machine learning

**Figure 3:**
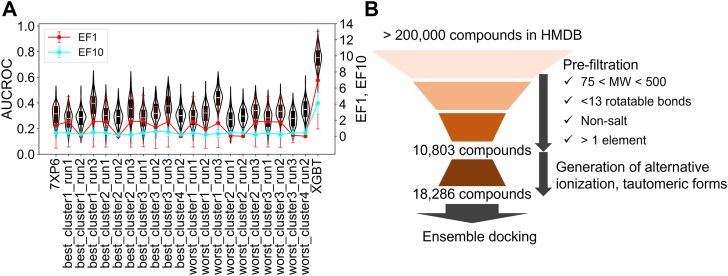
**Comparison of predictive performance and treatment workflow of compounds in HMDB.***A*, predictive performance of each model measured in nested cross-validation. The AUCROC values of each model are shown as violin plots, while the EF1 and EF10 values are represented by red and cyan points, respectively. Data are expressed as mean ± S.D. (N = 120, 30 × 4 nested-cv). *B*, Workflow for HMDB compounds prior to ensemble docking.

To optimize hyperparameters and evaluate predictive performance, we constructed a benchmark dataset using BitterDB (29) and the DeepCoy algorithm (30). Briefly, compounds meeting the criteria of an activation threshold of ≤10 μM and a molecular weight of ≤500 were selected as active compounds, and approximately 50 decoys were generated for each active compound based on its SMILES representation. These compounds in the benchmark dataset were docked to the sampled receptor structures, and their docking affinities were calculated. For the machine learning algorithm, we employed Extreme gradient boosting trees (XGBT), which demonstrated strong performance in previous *in silico* screening studies (26). Nested cross-validation was then conducted for hyperparameter optimization and model evaluation. The results showed that all evaluation metrics—AUCROC, EF1, and EF10—were improved when using XGBT compared with predictions from docking affinities based on single conformations (Fig. 3*A*, Table S1). The mean AUCROC achieved by XGBT was 0.75, whereas the best-performing single conformation yielded ∼0.44. Similarly, XGBT achieved EF1 and EF10 values of ∼6.97 and ∼4.11, respectively, compared with ∼1.82 and ∼0.62 for the best single-conformation results. These findings demonstrate that XGBT-based machine learning is effective for predicting TAS2R46 agonists.

**Figure 4:**
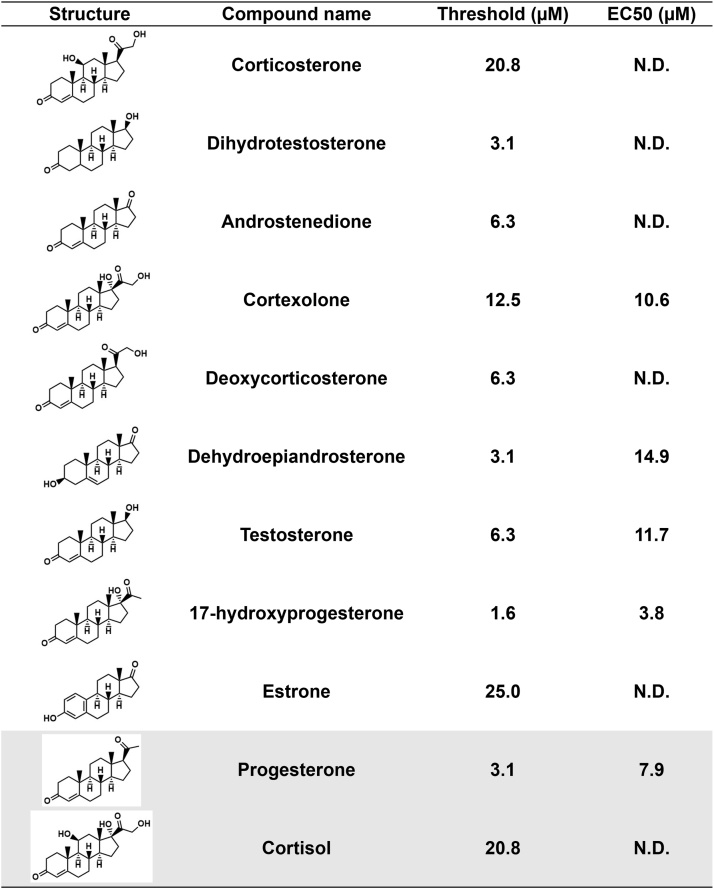
**List of candidate compounds for the subsequent TAS2R46 functional assay.** All candidate compounds are listed. The known TAS2R46 agonists, progesterone and cortisol, are shaded in gray. Threshold was defined as the lowest concentration at which RLU values first reached statistical significance (*p* < 0.05) compared with the minimum concentration tested (one-way ANOVA and Dunnett’s *post hoc* test). N.D.: not determined.

To prioritize the candidate agonists, compounds from the HMDB were preprocessed following the workflow shown in Figure 3*B*. Ensemble docking was then performed against these compounds, followed by application of the trained machine learning model. Predictions from this model were used to rank compounds by their predicted probabilities (Table S2). Among the HMDB entries, those classified as “Detected and Quantified” were prioritized because their concentrations have been experimentally confirmed in the human body. From this verified class, the final candidate agonists selected were steroid hormones and their derivatives, including progesterone and cortisol, both of which were previously reported as TAS2R46 agonists (Fig. 4) (5).

### TAS2R46 functional assays and interaction mode analysis

**Figure 5:**
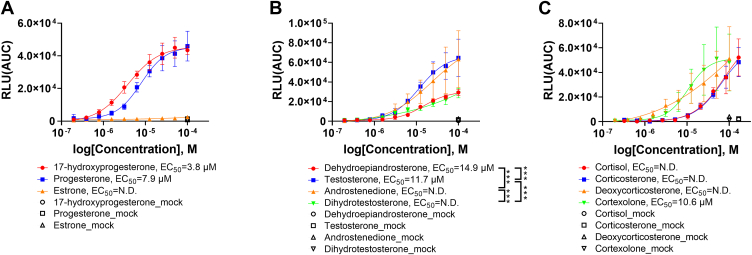
**Dose–response curves of TAS2R46 stimulation with candidate compounds in the luminescence assay.** To assess the TAS2R46 activation by the candidate compounds, HEK293 cells were transiently transfected with plasmids encoding TAS2R46, Gα16-gust44, and Clytin II, whereas cells transfected with Gα16-gust44 and Clytin II were used as mock-transfected controls. The dose–response curves are shown for (*A*) progestogens: progesterone, 17-hydroxyprogesterone, and estrone; (*B*) androgens: dehydroepiandrosterone, testosterone, androstenedione, and dihydrotestosterone; and (*C*) corticoids: cortisol, corticosterone, deoxycorticosterone, and cortexolone. Data are expressed as the mean ± S.D. All transfections were performed in triplicate; each experiment was independently repeated three times. N.D.: not determined. ∗∗∗*Ps* < 0.01, two-way ANOVA and Tukey’s *post hoc* test.

To validate the predictions, we conducted TAS2R46 functional assays using a heterologous expression system. First, we performed single-cell Ca^2+^ imaging to confirm the functionality of the system (Fig. S7). HEK293 cells transiently expressing TAS2R46 and Gα16-gust44 exhibited Ca^2+^ responses to corticosterone and testosterone, as well as to the known agonists andrographolide and progesterone. Next, we established a high-throughput luminescence-based assay to obtain concentration–response curves in HEK293 cells co-expressing TAS2R46 and Gα16-gust44 together with the luminescence protein Clytin II. All candidate compounds, except estrone, elicited concentration-dependent responses in TAS2R46-expressing HEK293 cells, whereas no detectable responses were observed in receptor-negative control cells (HEK293 cells expressing Gα16-gust44 and Clytin II in the absence of TAS2R46) (Figs. 5, S6 and S8). Progesterone and 17-hydroxyprogesterone, both belonging to the progestogens, exhibited higher potency than the other tested steroids, with EC_50_ values of 7.9 and 3.8 μM, respectively (*Ps* < 0.01, one-way ANOVA and Tukey’s *post hoc* test; Fig. 5*A*, Fig. S6, and Table S3). Androgens, testosterone and androstenedione, displayed significantly greater efficacy than the other androgens tested (*Ps* < 0.01, two-way ANOVA and Tukey’s *post hoc* test; Fig. 5*B* and Table S4). Within the mineralocorticoids and glucocorticoids, cortisol and corticosterone showed lower potency than their metabolic intermediates, deoxycorticosterone and cortexolone, respectively (Figs. 4 and 5*C*, Tables S5). Collectively, these results suggest that, consistent with our predictions, a variety of steroid hormones and their derivatives can activate TAS2R46, and that its potency and efficacy are influenced by the structural features of the compounds.

**Figure 6:**
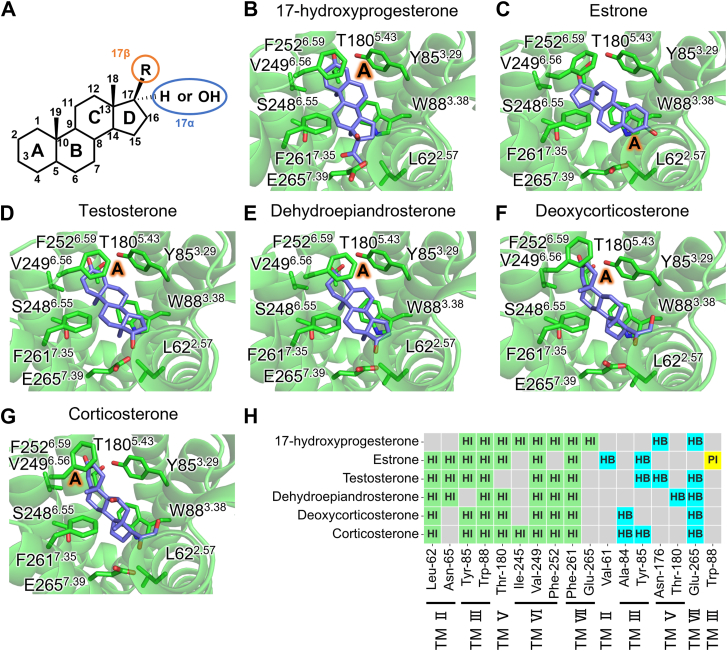
**Binding poses and interaction modes of representative candidates.***A*, Backbone of steroids. *B–F*, binding poses of representative candidates: (*B*) 17-hydroxyprogesterone, (*C*) estrone, (*D*) testosterone, (*E*) dehydroepiandrosterone, (*F*) deoxycorticosterone, and (*G*) corticosterone. Residues mediating interactions shared between TAS2R46 and the candidates, as well as those involved in hydrogen bonding, are shown as stick models. The A-ring of the steroid backbone is indicated by the letter “A”. *H*, the interaction modes of the candidates. Each interaction mode is represented by a color and abbreviation: hydrophobic interaction (HI, *green*), hydrogen bond (HB, *cyan*), and π interaction (PI, *yellow*).

To gain insight into the structure–activity relationship, we modeled complexes of TAS2R46 with representative ligands using Boltz-2 (31) (Fig. 6). Across all models, several hydrophobic interactions were commonly observed. In particular, interactions with Trp-88^3.32^ in transmembrane helix (TM) III, Val-249^6.56^ in TM VI, and Phe-261^7.35^ in TM VII were consistently detected. In addition, hydrophobic interactions with Leu-62^2.57^ in TM II, Tyr-85^3.29^ in TM III, Thr-180^5.43^ in TM V, and Phe-252^6.59^ in TM VI were observed in at least five complexes. Among the tested compounds, 17-hydroxyprogesterone, which exhibited the lowest EC_50_, is characterized by a 17α-hydroxy group (Figs. 4 and 6*A*). In its predicted binding pose, this 17α-hydroxy group was positioned near TM VI and TM VII, forming a hydrogen bond with Glu-265^7.39^ in TM VII (Fig. 6, *B* and *H*). In the binding pose of the non-agonist estrone, a distinctive interaction mode was observed; the A-ring was positioned near TM VII, formed π–π stacking interactions with Trp-88^3.39^, but did not interact with Glu-265^7.39^, in contrast to the other steroid compounds (Fig. 6, *C* and *H*). Analogously to 17-hydroxyprogesterone, testosterone, dehydroepiandrosterone, deoxycorticosterone, and corticosterone commonly formed a hydrogen bond with Glu-265^7.39^, while differences among them were observed in the interaction modes between their A-rings and residues in TM V (Fig. 6, *D*–*H*). The A-ring of testosterone formed a hydrogen bond with Asn-176^5.39^, whereas that of dehydroepiandrosterone formed a hydrogen bond with Thr-180^5.43^. Corticosterone and deoxycorticosterone did not show any interactions with the residues in TM V. Taken together, these results suggest that a hydrogen bond with Glu-265^7.39^ and hydrophobic interactions with residues such as Trp-88^3.32^ may contribute to ligand potency and efficacy.

**Figure 7:**
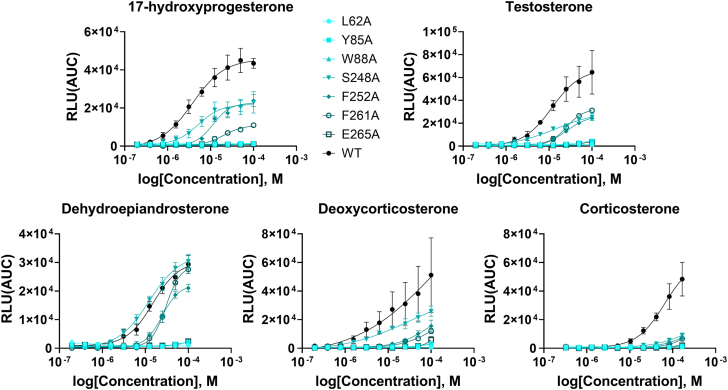
**Dose–response curves of TAS2R46 mutants stimulated with representative candidates in a luminescence assay.** Luminescence assays were performed using TAS2R46 mutants to assess the effects of alanine substitutions at residues predicted to be involved in ligand binding. The dose–response curves are shown for (*A*) 17-hydroxyprogesterone, (*B*) testosterone, (*C*) dehydroepiandrosterone, (*D*) deoxycorticosterone, and (*E*) corticosterone. Data are expressed as the mean ± S.D. All transfections were performed in triplicate; each experiment was independently repeated three times. Details of the statistical analysis were presented in Figs. S8, S9, and Tables S7–S10.

Next, we performed site-directed mutagenesis of residues predicted to interact with, or be in close proximity to, these steroid compounds and evaluated their effects on receptor activation. Alanine substitutions at Leu-62^2.57^ (L62A), Tyr-85^3.29^ (Y85A), Trp-88^3.32^ (W88A), and Glu-265^7.39^ (E265A) diminished activation by all tested compounds (Fig. 7). Mutants at Ser-248^6.55^ (S248A), Phe-252^6.59^ (F252A), and Phe-261^7.35^ (F261A) retained dose–response relationships, with at least one of efficacy or potency reduced for most compounds (Fig. 7). For 17-hydroxyprogesterone, testosterone, and dehydroepiandrosterone, for which EC_50_ values were determined (Table S7), the S248A mutation reduced the efficacy of 17-hydroxyprogesterone and testosterone but not that of dehydroepiandrosterone(Figs. 7, S8, and S9, and Tables S7–S10). In contrast, the S248A mutation did not affect the potency of any of these three compounds (Figs. 7, S8, and S9, and Tables S7–S10). The F252A mutation reduced both efficacy and potency for all three compounds (Figs. 7, S8, and S9, and Tables S7–S10). The F261A mutation showed a similar effect for 17-hydroxyprogesterone and testosterone, but for dehydroepiandrosterone, reduced potency while maintaining efficacy comparable to that of the WT (Figs. 7, S8, and S9, and Tables S7–S10). Collectively, these results suggest that these residues contribute to the stable binding of the steroid compounds and to the activation of TAS2R46.

## Discussion

Bitterness is one of the five basic tastes, and the sensing of it acts as a defense mechanism against potentially harmful substances. The expression of TAS2Rs in extra-oral tissues that are not directly exposed to the external environment suggests the existence of endogenous ligands and highlights their role as chemical sensors that monitor the internal environment. Among them, TAS2R46 is a receptor whose structure has been resolved and is reported to be expressed in various extra-oral tissues, including airway epithelial tissue (32), smooth airway muscle (33), sinonasal tissue (34), heart tissue (35), and skeletal muscle (36). Moreover, a consensus dataset integrating the Human Protein Atlas (https://www.proteinatlas.org) and GTEx transcriptomic datasets (https://www.proteinatlas.org/) shows that TAS2R46 is expressed in the cerebellum, pituitary gland, urinary bladder, testis, ovary, cervix, and adipose tissue. Identifying the endogenous ligands of TAS2R46 is therefore critical for elucidating its potential physiological roles in these extra-oral tissues.

In this study, we employed a strategy that combines ensemble docking with machine learning to select promising compounds, beginning with conformation sampling of TAS2R46 from MD trajectories. Conformational clustering around the binding site, followed by rescoring of strychnine binding affinity, enabled the selection of diverse conformations for subsequent docking (Fig. 2). Among the selected models, three major binding poses were identified, two of which differed from those in the experimental structure (Fig. S5). Despite these differences, strychnine interacted with Trp-88^3.32^ and Glu-265^7.39^, which are critical for its activation (23). Moreover, in the TAS2R46 structure (PDB ID: 7XP6), the electron density of strychnine appeared as a bulky and flattened sphere, allowing multiple possible orientations. Therefore, the binding poses observed in the selected models likely represent plausible alternative binding modes.

The aggregation of predicted binding affinities using machine learning improved the evaluation metrics (AUCROC, EF1, and EF10) compared with those obtained from individual conformations (Fig. 3*A*). Among the top 1000 compounds screened from the HMDB (28) (Table S2), many possessed a steroid backbone, with some steroid hormones and their sulfate conjugates appearing particularly frequently. TAS2R46 functional assays demonstrated that all tested compounds, except for estrone, elicited concentration-dependent responses. In addition to the two known TAS2R46 agonists, progesterone and cortisol (5), our virtual screening newly identified eight steroid hormones as TAS2R46 agonists, thereby validating the effectiveness of our ligand identification strategy. It remains possible that additional endogenous ligands of TAS2R46 are present among the top 1000 compounds that were not selected for the functional assay.

According to the HMDB, the blood concentrations of these compounds are less than 0.1 μM, which is below the minimum concentration we tested (EC_50_ of 3.8 μM for 17-hydroxyprogesterone). However, steroid hormones are primarily biosynthesized in the adrenal glands and the gonads, (testis and ovary), where their local concentrations can be substantially higher than in circulation. For example, intratesticular testosterone concentrations are more than 100-fold higher than in serum, exceeding 1 μM (37, 38). Furthermore, progesterone and 17-hydroxyprogesterone concentrations in follicular fluid reach tens of micromolar and several micromolar levels, respectively (39, 40, 41). Interestingly, TAS2R46 expression in testis and ovary was confirmed in the consensus dataset, as described above, and steroid hormone concentrations in these tissues are sufficient to activate TAS2R46. In addition, various peripheral tissues, including adipose tissue, skeletal muscle, liver, endometrium, prostate, skin, and salivary gland, express steroid-metabolizing enzymes that locally convert androgen precursors such as dehydroepiandrosterone and conjugated steroid hormones in circulation into more active forms, which are then utilized for tissue-specific biological processes (a mechanism termed ‘intracrine’) (42, 43, 44). Although steroid synthesis is primarily restricted to the intracellular compartment, membrane-permeable steroid hormones could be secreted and may achieve sufficiently high local concentrations to activate TAS2R46 in an autocrine and paracrine manner. Taken together, these findings suggest that TAS2R46 may function as a sensor of locally elevated steroid hormones, thereby contributing to tissue-specific steroid signaling beyond their classical systemic roles. Further studies are required to elucidate novel physiological roles of TAS2R46 expressed in extra-oral tissues.

In the present ligand screening, no clear correlation was observed among rank, potency, and efficacy. This may stem from the fact that the machine learning model was constructed for classification into active/inactive compounds based on threshold values, but no regression model was developed for predicting EC_50_ or continuous activity values. Furthermore, because the receptor structure used was specifically optimized for the strychnine binding pocket, the candidate compounds identified may have been subject to certain structural constraints, potentially limiting the diversity of functional outcomes.

Our predicted TAS2R46–ligand complexes provided insight into the structure–activity relationship (Fig. 6). All agonists consistently exhibited hydrophobic interactions with Trp-88^3.32^, a conserved residue across TAS2Rs and implicated in TAS2R46 activation (23), as well as with Val-249^6.56^, and Phe-261^7.35^. In addition, some agonists formed hydrophobic interactions with other residues in the binding pocket, including Leu-62^2.57^, Tyr-85^3.29^, Thr-180^5.43^, and Phe-252^6.59^. These observations suggest that common structural features of the agonists, such as the steroid backbone with 18- and 19-methyl groups, contribute to interactions with these hydrophobic residues, thereby stabilizing the binding. Mutagenesis analysis showed that alanine substitutions at Leu-62^2.57^, Tyr-85^3.29^, Trp-88^3.32^, Phe-252^6.59^, and Phe-261^7.35^ altered receptor activation by the identified steroid compounds. In particular, L62A, Y85A, and W88A, markedly reduced activation, whereas F252A and F261A decreased both efficacy and potency across the tested compounds. Together, the results indicate that these residues are critical for stable binding to steroid compounds and for receptor activation. In addition to these hydrophobic interactions, hydrogen-bonding interactions were also observed. With the exception of estrone, the steroid compounds formed a hydrogen bond with Glu-265^7.39^, and some additionally interacted with Asn-176^5.39^ and Thr-180^5.43^ in TM V. A previous study reported that Asn-176^5.39^ interacted with strychnine in MD simulations, and that its alanine substitution at Asn-176^5.39^ affects receptor responses *in vitro* (45). Moreover, the experimental structure of TAS2R46 indicates that Thr-180^5.43^ is positioned sufficiently close to strychnine to enable interaction (23). These findings suggest that differences in interaction patterns within TM V may contribute to ligand-specific responses. Glu-265^7.39^ has been reported to play a critical role in TAS2R46 activation and ligand specificity (4, 23). Notably, estrone, which is inactive, did not interact with Glu-265^7.39^, and the loss of activation by other agonists in the E265A mutant further supports the importance of the interaction between steroid agonists and Glu-265^7.39^ for receptor activation. Ser-248^6.55^ is located near the steroid agonists but did not show direct interactions in the predicted models (Fig. 6). Among the compounds for which EC_50_ values were determined—testosterone, 17-hydroxyprogesterone, and dehydroepiandrosterone—the S248A mutation reduced the efficacy of testosterone and 17-hydroxyprogesterone but did not affect the potency of any of the three compounds (Figs. 7 and S8). These results suggest that Ser-248^6.55^ may not be directly involved in ligand binding but may contribute to receptor activation, possibly by influencing conformational changes required for signaling.

In this study, we identified eight steroid hormones (including their derivatives) as potential endogenous ligands of TAS2R46 by combining ensemble docking of multiple conformations sampled from MD trajectories with machine learning, followed by functional assays. Furthermore, Boltz-2–based predictions of TAS2R46–steroid complexes and mutational analysis revealed that the steroid backbone interacts with crucial residues, leading to subsequent receptor activation. Thus, our study not only provides new insights into the physiological roles of TAS2R46 but also offers a broadly applicable strategy for exploring endogenous ligands across the GPCR superfamily, including other TAS2Rs.

### Limitations of this study

In this study, structure–activity relationships of the steroid hormones and their derivatives were estimated by their binding poses predicted by Boltz-2. Given the complex mechanisms of TAS2R46 activation beginning from its ligand recognition, further studies, including additional *in vitro* mutational analysis and more precise calculations such as MD simulations, are needed to elucidate the detailed structure–activity relationships of these compounds.

## Experimental procedures

### Construction of benchmark and HMDB compound datasets

To construct the benchmark dataset for assessing the accuracy of the screening methods, the csv file that contained hTAS2R46 active compounds was downloaded from BitterDB (29). The active compounds in the benchmark dataset were selected based on the following criteria: an activation threshold of 10 μM or less and a molecular weight of 500 or less. Additionally, the DeepCoy algorithm (30) was applied to generate decoys that have properties resembling those of the selected active compounds. 50 decoys per active compound were generated using DeepCoy. Scrubber (https://github.com/forlilab/molscrub) was also used to generate 3D conformers of the active compounds and decoys at pH 7.4 from SMILES, and then Meeko (https://github.com/forlilab/Meeko) was applied to store the information of 3D conformations in PDBQT format. The compounds that could not be constructed were removed. Ultimately, we compiled a benchmark set consisting of 2442 compounds with generated three-dimensional structures—48 active compounds and 2394 decoys (hit rate:∼2%).

To construct the dataset for the screening, a csv file containing the data of all metabolites was downloaded from HMDB in XML format. From the data of all metabolites, over 10,000 compounds were selected based on the following criteria: a rotatable bond count of 12 or less, a molecular weight between 75 and 500, not being salts, and not being composed solely of a single atom. Scrubber and Meeko were used for generating 3D conformers of protomers and tautomers at pH 7.4 from SMILES. These conformer data were stored in PDBQT format, and the total number of compounds in the dataset was 18,286.

### Molecular modeling

The general numbering of residues was based on the primary sequence of TAS2R46. Superscripted residue numbers in TAS2R46 follow the generic numbering system of Ballesteros and Weinstein (46).

The structures of hTAS2R46 were obtained from the PDB and AlphaFold Database (19, 47), using the PDB ID 7XP6 (23) and the AlphaFold model AF_AFP59540F1. In the cryo-EM structure of activated TAS2R46 (7XP6), several residues are missing, and limited resolution in the orthosteric binding site has been reported previously (48). In particular, the side chains of Glu-265^7.39^, Asn-176^5.39^, and Phe-261^7.35^ were assigned in regions lacking clear electron density. To address these limitations, we used a TAS2R46 structure predicted by AlphaFold2, which contains no missing residues, and performed MD simulations to sample diverse conformations. The RMSD of Cα atoms between the AlphaFold2 model and the experimental structure was 1.144 Å (Fig. S10). The C-terminal region (302–309) was removed due to its low predicted local distance difference test (pLDDT) score and its absence in the experimental structure. The structure of strychnine was obtained from PDB (Ligand ID: SY9). For conformational sampling, strychnine was docked into the AlphaFold2 model using AutoDock Vina (49).

### System setup

The hTAS2R46–strychnine complex in the absence of G-protein was inserted into a pre-equilibrated POPC bilayer using the membrane builder on the CHARMM-GUI web server (www.charmm-gui.org) (50, 51, 52). The restrained ESP (RESP) charges of the ligands were generated by the antechamber program implemented in Amber24 (53). AMBER ff19SB (54), lipid21 (55), and general AMBER force field 2 (56) were used for proteins, lipids, and ligands, respectively. Each system was solvated in the OPC water model (57) and neutralized with Na^+^/Cl^−^ (150 mM).

### Molecular dynamics simulation

All simulations were conducted under periodic boundary conditions. The short-range cut-off was 9 Å for unbonded interactions. Long-range electrostatic interactions were calculated by the Particle Mesh Ewald (PME) summation method. Simulations were performed using Amber24 for both the equilibration and production processes, together with the SHAKE algorithm (58) based on the protocols provided by the CHARMM-GUI web server. As the first step of equilibration processes, energy minimization was performed using the steepest descent method for 2500 steps, followed by the conjugated gradient method for 2500 steps. Subsequently, the systems were heated to 310.0 K, and an NVT (constant particle number, volume, and temperature) simulation was performed with a 1-fs time step for 250 ps. Then, an NPT (constant particle number, pressure, and temperature) simulation was carried out with a 1-fs time step for 0.125 ns and a 2-fs time step for 1.5 ns. Thereafter, production runs were performed with a 2-fs time step for 500 ns. Three independent simulations were conducted for each experiment. The equilibrium states of the simulated models were assessed by calculating the root-mean-square deviation (RMSD) of the Cα structure of TAS2R46 using MDAnalysis (59, 60).

### Selection of representative conformations

As a first step, to characterize each conformation, the residues relevant to ligand recognition were defined as those located within 5 Å of strychnine for more than 30% of the concatenated 1500-ns MD trajectory (Fig. S1). To select the hTAS2R46 conformations from the MD simulation trajectories of strychnine–hTAS2R46, the align module in MDAnalysis (59, 60) was implemented to compute a pairwise RMSD matrix based on 24 residues selected above over the last 300 ns of each MD run, during which the Cα RMSD had stabilized (Fig. S2). Subsequently, the pairwise RMSD matrix was used to perform Ward clustering, and the number of clusters was decided based on the reduced Cartesian space displayed by PCA, in addition to the dendrograms (Fig. S3). Thereafter, the binding affinity of strychnine in each frame was estimated by the Vina scoring function implemented in GNINA 1.0 (61), and then the highest- and lowest-affinity conformations in each cluster were selected for the following ensemble docking. The modes of interaction between strychnine and TAS2R46 were analyzed using the Protein-Ligand Interaction Profiler (PLIP) (62).

### Ensemble docking

All of the selected conformations and the experimental structure 7XP6 were stored as PDBQT files using MGLtools1.5.6 (63). Ensemble docking was performed using Uni-Dock (64) based on the following settings: the dimensions of the grid box set to 20 Å for length, width, and height; the scoring function set to Vinardo (65), a fork of Vina; and the search mode set to “detail.”

### Machine learning

XGBT, a machine learning algorithm, was implemented using the XGBoost library (66) to develop target-specific binary classifiers to identify potential active molecules from among the decoys. The performance of XGBT was evaluated by 30 repetitions of nested 4-fold cross-validation (30 × 4 nested-cv). At each nested repetition, the benchmark dataset was stratified 4-fold, with 75% (3-fold) used for model building and hyperparameter optimization by stratified 4-fold cross-validation. In the inner cross-validation, 25% of 75% (3-fold) was used for early stopping. Optuna (67) was used for the hyperparameter optimization, and the evaluation metric was the area under the curve of the receiver operator characteristic (AUCROC). Finally, the optimized model constructed in the inner loop was evaluated using the rest of the outer loop (initial 25%) and based on the predicted probability of each compound. We used AUCROC and Enrichment Factor (EF) as metrics for evaluating screening performance. EF indicates how many more active compounds are present within the top X fraction compared with what would be expected by random sampling (68). EF is calculated by the following equation.

$$EFX=\frac{{Active}_{TopX\%}/{Total}_{TopX\%}}{{Active}_{dataset}/{Total}_{dataset}}$$

Here, *Active*_*TopX%*_ and *Total*_*TopX%*_ are the numbers of active compounds and total compounds in the top X% in the compound list ordered by rank, respectively, while *Active*_*datdaset*_ and *Total*_*dataset*_ are the numbers of active compounds and total compounds in the benchmark dataset. The combination of hyperparameters that achieved the best AUCROC was used for subsequent predictions (Table S1). Additionally, we also calculated the AUCROC, EF1, and EF10 values from the initial 25% in the outer loop of the raw docking data as measures of performance of each conformation.

### Screening of candidate compounds

Candidate compounds from HMDB were filtered using the following criteria: a molecular weight between 75 and 500, fewer than 13 rotatable bonds, not being a salt, and containing more than one element. Then, ensemble docking was performed using the constructed machine learning model to narrow down the candidate compounds. As a result of prediction by machine learning, these compounds were ranked by predicted probability (Table S2). HMDB classified the compounds into four classes: “Quantified,” “Detected but not Quantified,” “Expected,” and “Predicted.” (28). The first of these, “Quantified,” indicates that compound concentrations in the human body were determined experimentally. Therefore, based on the predicted ranking, the final candidate compounds were selected from the HMDB compounds classified as “Quantified.”

### Prediction of TAS2R46–candidate complexes by Boltz-2

Boltz-2 (31) was locally installed and its calculations were performed by Genkai, a supercomputer at the Research Institute for Information Technology, Kyushu University. The structure predictions were conducted using the sequence of amino acids 3 to 301 of TAS2R46, corresponding to the region resolved in the experimental structure (PDB ID: 7XP6). The candidates were specified by the following PDB CCD codes: 17-hydroxyprogesterone (3QZ), estrone (J3Z), testosterone (TES), deoxycorticosterone (1CA), and corticosterone (C0R). Multiple sequence alignments were generated using Colabfold (69). Then, mmCIF information of the three experimental structures was inserted into the template section of each JSON file: TAS2R46 (PDB ID: 7XP6), TAS2R14 (PDB ID: 8VY7), and TAS2R16 (PDB ID: 9K6L). The complexes of TAS2R46 and each candidate were generated using the following the optional settings: “recycling_steps = 10”, “diffusion_samples = 10”, “diffusion_samples_affinity = 10”, “use_potentials = True”, “subsample_msa = True”, and “num_subsampled_msa = 1024”. In the complex of each candidate, energy minimization against the candidate and the residues within 5 Å of it was performed using the steepest descent method for 100 steps, followed by the conjugated gradient method for 10 steps by UCSF Chimera (70). The modes of interaction between TAS2R46 and each compound were analyzed by PLIP (62).

### Preparation of expression constructs

Constructs encoding human TAS2R46 (Accession ID: NM_176887.2), the chimeric G protein α subunit Gαl6-gust44 and the photoprotein, Clytin II, were cloned into the pEF-DEST51 Gateway vector (Life Technologies, Carlsbad, CA, USA) (71, 72). A Kozak cassette was introduced at the 5′ end before the start codon. To improve the transportation of TAS2R46 to the plasma membrane, the coding sequence for the first 45 amino acids of SST3 was fused with the start codon of *TAS2R46*. Clytin II was targeted to mitochondria by fusing the coding sequence for the first 29 amino acids of cytochrome c oxidase (subunit VIII) with its start codon, as described previously (73). Site-directed mutagenesis (Takara Bio Inc., Shiga, Japan) was used to introduce point mutations into TAS2R46. DNA sequencing was performed to confirm the integrity of all of the DNA constructs.

### Functional expression

HEK293 cells (kindly provided by Dr Makoto Tominaga, Nagoya City University, Japan) were cultured in Dulbecco’s modified Eagle’s medium supplemented with 10% fetal bovine serum at 37 °C under a humidified atmosphere including 5% CO_2_. HEK293 cells were transiently transfected using Lipofectamine 2000 transfection reagent (Thermo Fisher Scientific, Waltham, MA, USA; 2.5 μl per 0.9 μg DNA). For single-cell Ca^2+^ imaging, cells were transfected with 0.9 μg of plasmids encoding TAS2R46 and Gα16-gust44. For luminescence assays, cells were transfected with 4.5 μg of plasmids encoding TAS2R46 and Gα16-gust44, together with Clytin II. Cells were seeded into 35-mm chambers (ibidi, Martinsried, Germany) at a density of 2,000,000 cells/well for single-cell calcium imaging, or into 384-well white plates with clear flat bottoms (Corning) at a density of 20,000 cells/well for luminescence assays. Gα16-gust44, a chimeric Gα protein in which the C-terminal 44 amino acids of Gα16 are replaced with those of gustducin, was employed to directly couple TAS2Rs to intracellular calcium signaling (6, 74). Clytin II was used as a luminescent reporter to monitor intracellular calcium mobilization upon receptor activation. As mock-transfected controls, HEK293 cells were transiently transfected with plasmids encoding Gα16-gust44 alone (for single-cell Ca^2+^ imaging) or Gα16-gust44 together with Clytin II (for luminescence assay).

### Single-cell Ca^2+^ imaging

Fluorescence-based Ca^2+^ imaging was conducted as previously described (75). At 24 h post-transfection, cells were incubated with 3.0 mM fluo-4 acetoxymethyl ester (Thermo Fisher Scientific) for 30 min at 37 °C. After loading, cells were rinsed with Hanks’ balanced salt solution (HBSS) supplemented with 10 mM HEPES. Test solutions prepared in HBSS containing 10 mM HEPES were delivered sequentially to the cells for 25 s at a flow rate of 1.0 ml/min using a peristaltic pump. Fluorescence signals were acquired using either an S Fluor 620/0.75 objective lens (Nikon, Tokyo, Japan) with a cooled charge-coupled device camera (C6790, Hamamatsu Photonics, Shizuoka, Japan) mounted on a TE300 microscope (Nikon), or a UPlanXApo 20 × /0.80 objective lens (Olympus) combined with an sCMOS camera (Zyla, ANDOR) on an IX73 microscope (Olympus). Image acquisition and subsequent analyses were carried out using AquaCosmos 1.3 (Hamamatsu Photonics) or CellSens Dimension 4.1 (Olympus). To minimize receptor desensitization, a 5-min interval was maintained between successive stimulations. Changes in intracellular Ca^2+^ levels were measured in individual responsive cells and expressed as relative fluorescence changes (ΔF/F_0_), calculated as (F − F_0_)/F_0_, where F_0_ denotes baseline fluorescence.

### Luminescence assay

The luminescence assay was performed in accordance with a previously reported method (73). Twenty-four hours after transfection, the cells were washed with HBSS containing 10 mM 4-(2-hydroxyethyl)-1-piperazineethanesulfonic acid (HEPES; pH 7.4). Then, the cells were loaded with coelenterazine loading buffer (HBSS containing 10 μM coelenterazine, pH 7.4) for 4 h at 37 °C in the dark. After 25 s of baseline reading, an aliquot of the luminescence assay buffer supplemented with 3 × ligand was added, and the light emission was recorded using a FlexStation three microplate reader (Molecular Devices Co) for an additional 65 s. The changes in luminescence intensity were monitored every 5.1 s. The response from each well was expressed as relative light units (RLU) and calculated using the AUC. The responses were averaged from at least three wells receiving the same stimulus.

### Solutions

Solutions were dissolved in dimethyl sulfoxide (DMSO) and then diluted in HBSS containing 10 mM HEPES to reduce the DMSO concentration to a maximum of 1.0% in the final experiments. The reagents were purchased from Tokyo Chemical Industry Co., Ltd, Tokyo, Japan (andrographolide, progesterone, 17-hydroxyprogesterone, androstenedione, dihydrotestosterone, deoxycorticosterone, cortexolone, and estrone), Nacalai Tesque, Inc (isoproterenol), and FUJIFILM Wako Pure Chemical Corporation (testosterone, cortisol, and corticosterone).

### Statistics and reproducibility

All dose–response relationships were determined by three independent experiments (biological replicates) performed in triplicate (technical replicates). Half-maximal effective concentration (EC_50_) values were calculated from individual concentration–response data using the four-parameter logistic curve fit of GraphPad Prism (GraphPad Software). TAS2R46 responses were analyzed using one-way (the effects of compounds on logEC_50_) or two-way (the effects of compounds and concentration) ANOVA followed by the Tukey–Kramer test. Normality was assessed using the Shapiro–Wilk test. Given the small sample size, normality tests have limited statistical power; however, no obvious deviations from normality were observed. Threshold was defined as the lowest concentration at which RLU values first reached statistical significance (*p* < 0.05) compared with the minimum concentration tested (one-way ANOVA and Dunnett’s *post hoc* test).

## Data availability

The data supporting the findings of this study are available from the corresponding authors upon reasonable request.

## Supporting information

This article contains supporting information.

## Conflict of interest

The authors declare that they have no conflicts of interest with the contents of this article.
