# Supplementary material for: Structure-based virtual screening identifies potential endogenous ligands of the human bitter taste receptor TAS2R46
Source: J Biol Chem. 2026 Jun 25;302(8):113295. doi: 10.1016/j.jbc.2026.113295 (PMC13393665; doi:10.1016/j.jbc.2026.113295)
Supplement: Supporting Figures and Tables [file mmc1.docx]

**Supporting Information for:**

Structure-based virtual screening identifies potential endogenous ligands of the human bitter taste receptor TAS2R46

Yuki Nagasato, Keisuke Sanematsu, Yuko Kawabata, Shingo Takai, and Noriatsu Shigemura

**The Supporting Information contains**

**Figures S1–10**

**Tables S1,3–10**


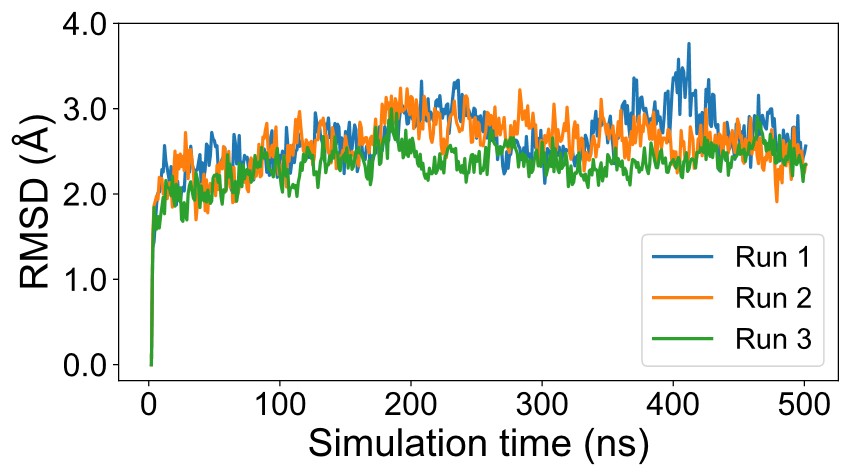


**Figure S1: Root-mean-square deviation (RMSD) of the Cα structure during molecular dynamics simulations of strychnine binding.** The plots show the RMSDs of the Cα structure of TAS2R46.


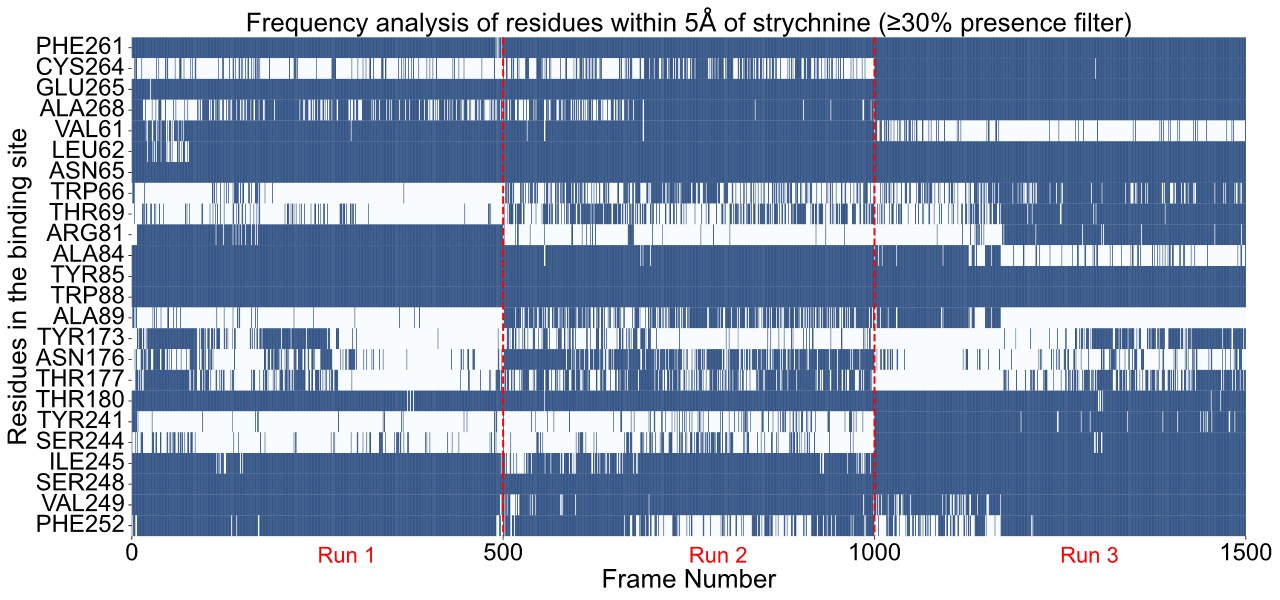


**Figure S2: Frequency analysis of residues within 5 Å of strychnine.**

Residues that were within 5 Å of strychnine for more than 30% of the concatenated trajectory are shown. Blue bars indicate residues within 5 Å of strychnine, while white bars indicate residues that were further away.


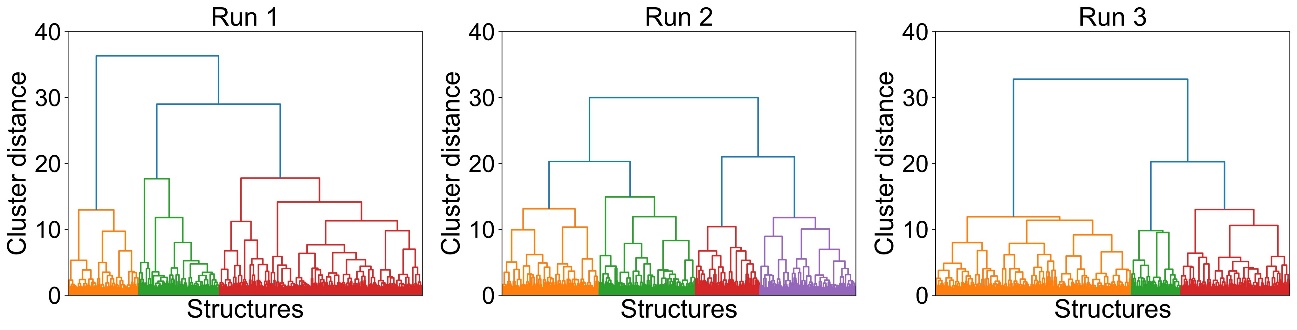


**Figure S3: Dendrograms from Ward clustering of each MD trajectory.**

The dendrograms were generated by Ward clustering analysis. Dendrograms corresponding to each MD run (runs 1, 2, and 3) are shown.


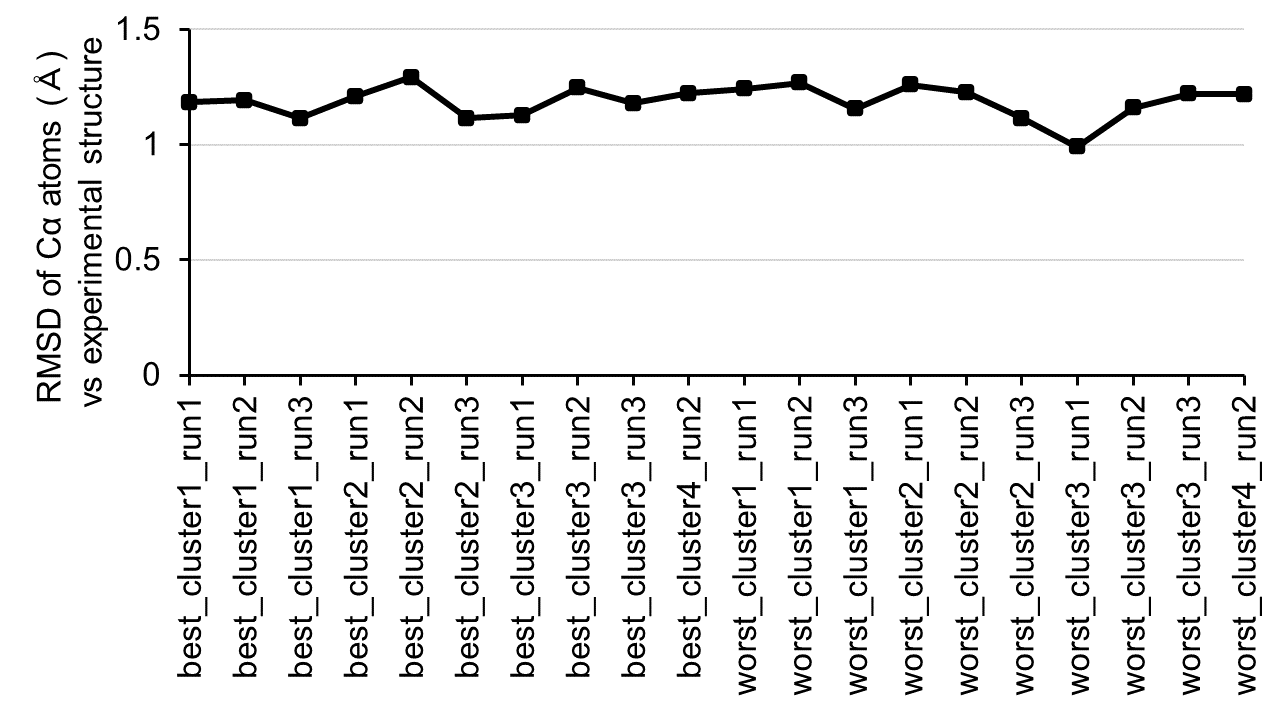


**Figure S4: Cα RMSD of the selected 20 structures relative to the experimental structure (7XP6).** The RMSD values were within the range of 0.99 Å to 1.29 Å.


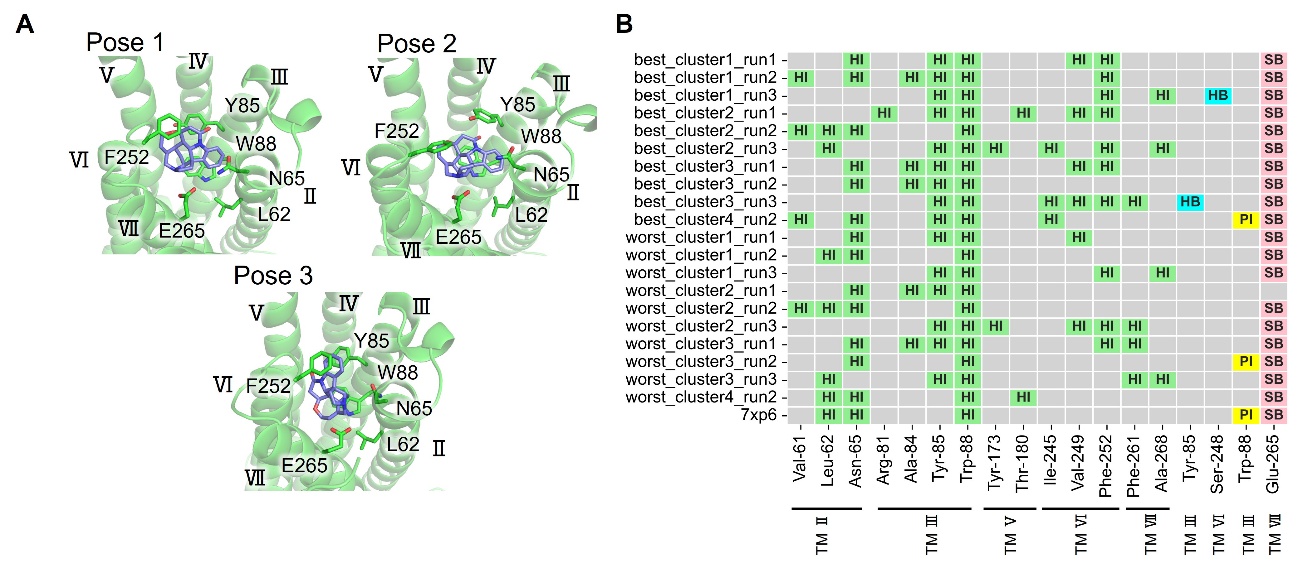


**Figure S5: Binding poses and interaction modes in the selected models.** (A) Binding poses of strychnine predominantly observed in the selected models. Strychnine and the main interacting residues are shown as purple and green sticks, respectively. The backbone of TAS2R46 is shown as a cartoon. (B) Interaction modes in the selected models. Each interaction mode is indicated by the color and abbreviation: hydrophobic interaction (HI, green), hydrogen bond (HB, cyan), π interaction (PI, yellow), and salt bridge (SB, pink).


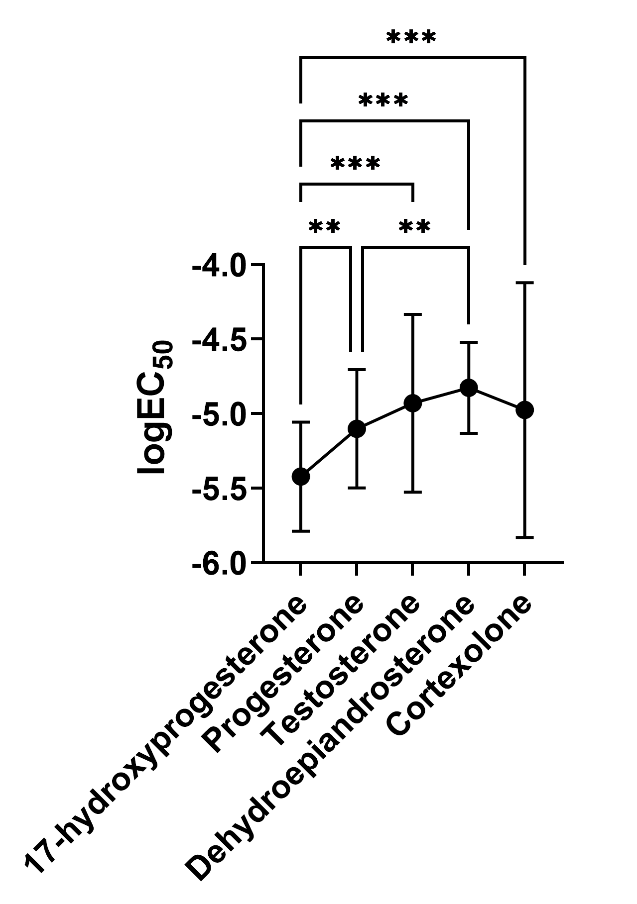


**Figure S6:** **Comparison of logEC_50_ values of the agonists.** Data are expressed as the mean ± S.D. **P < 0.01, ***P < 0.001 (one-way ANOVA and Tukey’s post hoc test). The figure is based on data shown in Figure 5.


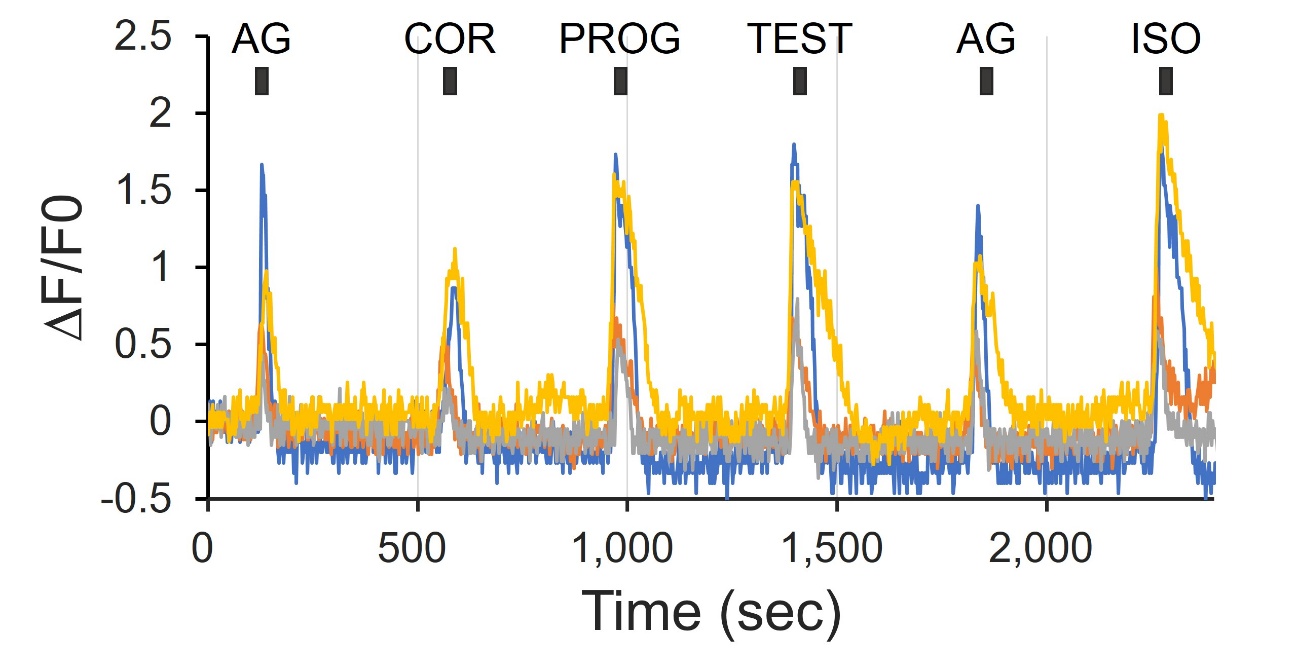


**Figure S7: Representative single-cell Ca²⁺ imaging traces obtained by fluorescence-based measurement.** HEK293 cells were transiently transfected with plasmids encoding TAS2R46 and Gα16-gust44, and intracellular Ca²⁺ levels were monitored using Fluo-4 AM fluorescence. Each trace represents the fluorescence intensity from an individual cell. The timing of compound application is indicated by black bars and the following abbreviations: andrographolide (AG, 100 µM), corticosterone (COR, 100 µM), progesterone (PROG, 100 µM), testosterone (TEST, 100 µM), and isoproterenol (10 µM). Andrographolide and progesterone are known agonists of TAS2R46.


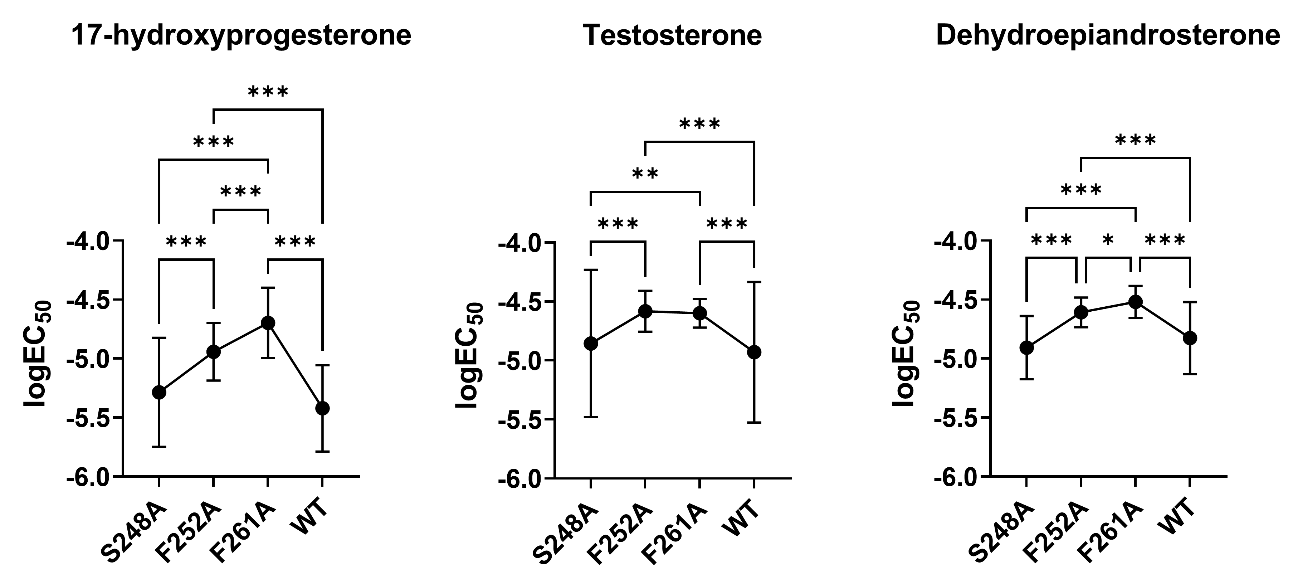


**Figure S8: Comparison of logEC_50_ values between the wild type (WT) and mutants for each agonist.** Data are expressed as the mean ± S.D. *P < 0.05, **P < 0.01, ***P < 0.001 (one-way ANOVA and Tukey’s post hoc test). The figure is based on data shown in Figure 7.


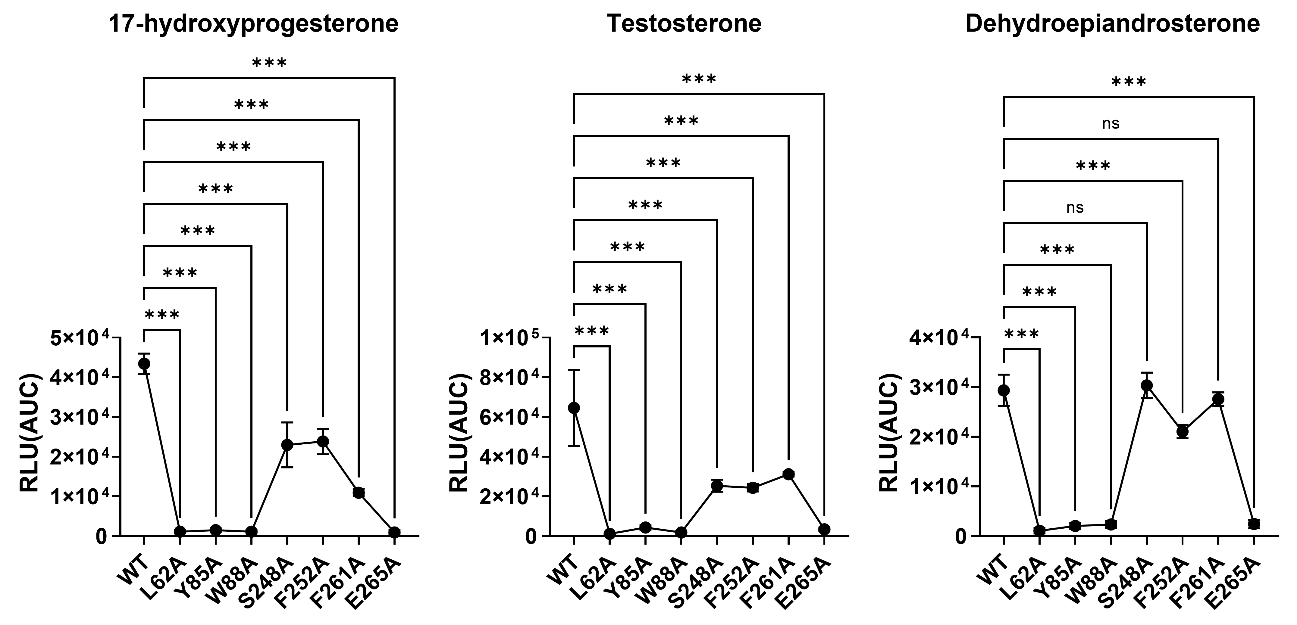


**Figure S9: Comparison of RLU values at the maximum concentration between the wild type (WT) and mutants for each agonist.** Data are expressed as the mean ± S.D. **P < 0.01, ***P < 0.001 (one-way ANOVA and Dunnett post hoc test). ns: not significant. The figure is based on data shown in Figure 7.


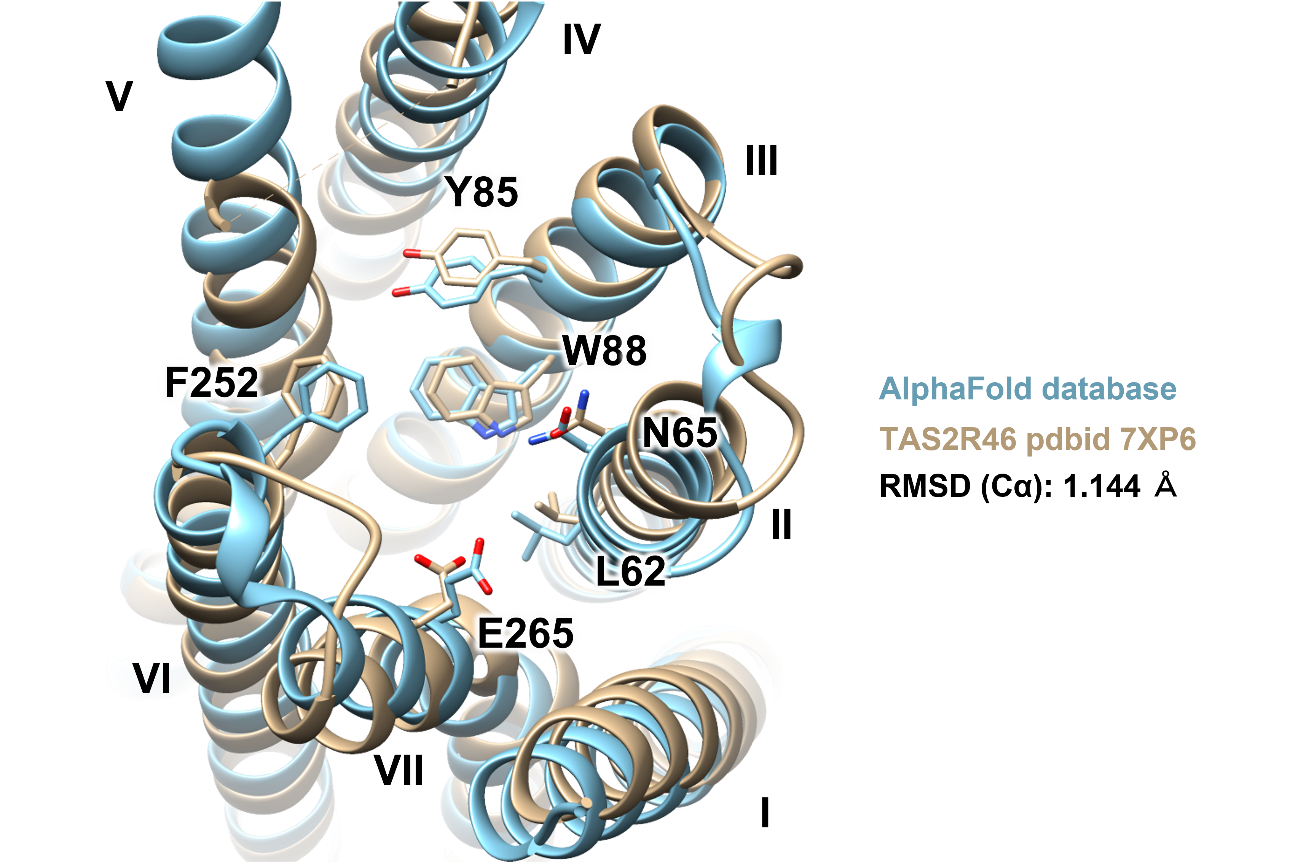


**Figure S10: Superposition of the AlphaFold database model onto the experimental structure (7XP6).** The AlphaFold database model used for the MD simulations was superposed onto the experimental structure (7XP6). Key residues involved in strychnine binding are shown as sticks.

**Table S1: The best hyperparameter setting of XGBT.**

| Hyperparameter name | Values |
| --- | --- |
| “objective” | “binary:logistic” |
| “booster” | “gbtree” |
| “colsample_bytree” | 0.1 |
| “device” | “cuda” |
| “gamma” | 0.017149 |
| “learning_rate” | 0.01 |
| “max_depth” | 2 |
| “min_child_weight” | 5 |
| “n_estimators” | 1038 |
| “random_state” | 42 |
| “reg_alpha” | 0.2738 |
| “reg_lambda” | 0.03195 |
| “scale_pos_weight” | 50 |
| “subsample” | 0.266428 |
| “tree_method” | “approx” |

**Table S3:** **Comparison of logEC_50_ among 17-hydroxyprogesterone, progesterone, testosterone, dehydroepiandrosterone, and cortexolone.**

| logEC_50_ | |
| --- | --- |
| Degrees of freedom | *F* value |
| 4, 430 | 15.60*** |

The effect of compounds was analyzed by one-way ANOVA. ****P* < 0.001. The table is based on data shown in Figure 5.

**Table S4:** **Comparison of the intracellular calcium responses of TAS2R46 to androgens: dehydroepiandrosterone, testosterone, androstenedione, and dihydrotestosterone.**

| Concentration | |  | Compound | |  | Compound × concentration | |
| --- | --- | --- | --- | --- | --- | --- | --- |
| Degrees of freedom | *F* value |  | Degrees of freedom | *F* value |  | Degrees of freedom | *F* value |
| 9, 320 | 149.4*** |  | 3, 320 | 72.17*** |  | 27, 320 | 8.549*** |

The effect of compounds was analyzed by two-way ANOVA. ****P* < 0.001. The table is based on data shown in Figure 7.

**Table S5: The Comparison of RLU values among the tested concentrations in each compound.**

| Compound | Degrees of freedom | *F* value |
| --- | --- | --- |
| 17-hydroxyprogesterone | 9, 80 | 203.5*** |
| Progesterone | 9, 80 | 122.0*** |
| Estrone | 9, 80 | 6.374*** |
| Androstenedione | 9, 80 | 23.67*** |
| Testosterone | 9, 80 | 79.52*** |
| Dehydroepiandrosterone | 9, 80 | 324.6*** |
| Dihydrotestosterone | 9, 80 | 114.3*** |
| Cortisol | 9, 80 | 70.44*** |
| Corticosterone | 9, 80 | 111.4*** |
| Deoxycorticosterone | 9, 80 | 17.72*** |
| Cortexolone | 9, 80 | 20.52*** |

The effect of compounds was analyzed by one-way ANOVA. ****P* < 0.001. The table is based on data shown in Figure 4 and 5.

**Table S6: The EC_50_ (µM) values of tested compounds.**

| Compound | WT | S248A | F252A | F261A |
| --- | --- | --- | --- | --- |
| 17-hydroxyprogesterone | 3.8 | 5.2 | 11.4 | 20.1 |
| Testosterone | 11.7 | 14.0 | 26.2 | 25.1 |
| Dehydroepiandrosterone | 14.9 | 12.4 | 24.7 | 30.2 |

The table is based on data shown in Figure7.

**Table S7: Comparison of logEC50 among S248A, F252A, F261A, and the wild type (WT) for each agonist.**

| logEC50 | | |
| --- | --- | --- |
| Compound name | Degrees of freedom | *F* value |
| 17-hydroxyprogesterone | 3, 344 | 75.98*** |
| Testosterone | 3, 344 | 13.83*** |
| Dehydroepiandrosterone | 3, 344 | 57.68*** |

The effect of compounds was analyzed by one-way ANOVA. ****P* < 0.001. The table is based on data shown in Figure7.

**Table S8: Comparison of the intracellular calcium responses of S248A, F252A, F261A, and the wild type (WT) across the agonists.**

|  | Concentration | |  | Genotype | |  | Genotype x Concentration | |
| --- | --- | --- | --- | --- | --- | --- | --- | --- |
| Compound name | Degrees of freedom | *F* value |  | Degrees of freedom | *F* value |  | Degrees of freedom | *F* value |
| 17-hydroxyprogesterone | 9, 640 | 494.3*** |  | 7, 640 | 1580*** |  | 63, 640 | 107.8*** |
| Testosterone | 9, 640 | 376.0*** |  | 7, 640 | 605.2*** |  | 63, 640 | 72.27*** |
| Dehydroepiandrosterone | 9, 640 | 1622*** |  | 7, 640 | 1570*** |  | 63, 640 | 231.8*** |
| Deoxycorticosterone | 9, 640 | 78.61*** |  | 7, 640 | 191.3*** |  | 63, 640 | 15.94*** |
| Corticosterone | 9, 640 | 219.2*** |  | 7, 640 | 483.0*** |  | 63, 640 | 90.49*** |

The effect of compounds was analyzed by two-way ANOVA. ****P* < 0.001. The table is based on data shown in Figure7.

**Table S9: Post-hoc comparisons among variants (including the wild type (WT)) for each agonist: 17-hydroxyprogesterone (17-OHP), Testosterone (T), dehydroepiandrosterone (DHEA), Deoxycorticosterone (DOC), and Corticosterone (CORT).**

|  | Adjusted p-value | | | | |
| --- | --- | --- | --- | --- | --- |
|  | 17-OHP | T | DHEA | DOC | CORT |
| L62A vs. Y85A | 0.9989 | 0.9998 | 0.8477 | 0.9991 | 0.9305 |
| L62A vs. W88A | 0.3744 | 0.2474 | 0.7141 | >0.9999 | 0.8577 |
| L62A vs. S248A | <0.0001 | <0.0001 | <0.0001 | <0.0001 | <0.0001 |
| L62A vs. F252A | <0.0001 | <0.0001 | <0.0001 | <0.0001 | 0.3859 |
| L62A vs. F261A | <0.0001 | <0.0001 | <0.0001 | 0.0182 | 0.9969 |
| L62A vs. E265A | 0.8916 | 0.9627 | 0.9995 | 0.9481 | 0.7472 |
| L62A vs. WT | <0.0001 | <0.0001 | <0.0001 | <0.0001 | <0.0001 |
| Y85A vs. W88A | 0.7698 | 0.5219 | >0.9999 | >0.9999 | >0.9999 |
| Y85A vs. S248A | <0.0001 | <0.0001 | <0.0001 | <0.0001 | <0.0001 |
| Y85A vs. F252A | <0.0001 | <0.0001 | <0.0001 | 0.0004 | 0.0192 |
| Y85A vs. F261A | <0.0001 | <0.0001 | <0.0001 | 0.1003 | 0.5464 |
| Y85A vs. E265A | 0.9964 | 0.9986 | 0.9863 | 0.9993 | >0.9999 |
| Y85A vs. WT | <0.0001 | <0.0001 | <0.0001 | <0.0001 | <0.0001 |
| W88A vs. S248A | <0.0001 | <0.0001 | <0.0001 | <0.0001 | <0.0001 |
| W88A vs. F252A | <0.0001 | <0.0001 | <0.0001 | 0.0001 | 0.0099 |
| W88A vs. F261A | <0.0001 | <0.0001 | <0.0001 | 0.0551 | 0.4149 |
| W88A vs. E265A | 0.9903 | 0.8909 | 0.9488 | 0.9942 | >0.9999 |
| W88A vs. WT | <0.0001 | <0.0001 | <0.0001 | <0.0001 | <0.0001 |
| S248A vs. F252A | <0.0001 | <0.0001 | <0.0001 | <0.0001 | 0.0733 |
| S248A vs. F261A | <0.0001 | <0.0001 | <0.0001 | <0.0001 | 0.0004 |
| S248A vs. E265A | <0.0001 | <0.0001 | <0.0001 | <0.0001 | <0.0001 |
| S248A vs. WT | <0.0001 | <0.0001 | <0.0001 | <0.0001 | <0.0001 |
| F252A vs. F261A | <0.0001 | 0.0874 | <0.0001 | 0.763 | 0.8361 |
| F252A vs. E265A | <0.0001 | <0.0001 | <0.0001 | 0.0038 | 0.0047 |
| F252A vs. WT | <0.0001 | <0.0001 | <0.0001 | <0.0001 | <0.0001 |
| F261A vs. E265A | <0.0001 | <0.0001 | <0.0001 | 0.3359 | 0.2909 |
| F261A vs. WT | <0.0001 | <0.0001 | <0.0001 | <0.0001 | <0.0001 |
| E265A vs. WT | <0.0001 | <0.0001 | <0.0001 | <0.0001 | <0.0001 |

**Table S10: Comparison of RLU values at the maximum concentration among S248A, F252A, F261A, and the wild type (WT) for each agonis.**

| RLU values at the maximum concentration | | |
| --- | --- | --- |
| Compound name | Degrees of freedom | *F* value |
| 17-hydroxyprogesterone | 7, 64 | 90.64*** |
| Testosterone | 7, 64 | 350.3*** |
| Dehydroepiandrosterone | 7, 64 | 613.9*** |

The effect of compounds was analyzed by one-way ANOVA. ****P* < 0.001. The table is based on data shown in Figure7.
